# Phytochemical Screening and Monitoring of Intercellular Changes in Murine Leydig Cells After the Treatment of *Trigonella foenum-graecum L*. Microgreens In Vitro

**DOI:** 10.33549/physiolres.935484

**Published:** 2025-02-01

**Authors:** Tomas JAMBOR, Zofia GOC, Lucia ZUSCIKOVA, Hana GREIFOVA, Anton KOVACIK, Eva KOVACIKOVA, Martin PEC, Norbert LUKAC

**Affiliations:** 1 Faculty of Biotechnology and Food Sciences, Institute of Applied Biology, Slovak University of Agriculture in Nitra, Nitra, Slovak Republic; 2 Faculty of Exact and Natural Sciences, University of the National Education Commission, Krakow, Krakow, Republic of Poland; 3 Faculty of Agrobiology and Food Resources, Institute of Nutrition and Genomics, AgroBioTech Research Centre, Slovak University of Agriculture, Nitra, Slovak Republic; 4 Slovak University of Agriculture in Nitra, Nitra, Slovak Republic; 5 Jessenius Faculty of Medicine in Martin, Institute of Medical Biology, Comenius University Bratislava, Martin, Slovak Republic

**Keywords:** Microgreens, *Trigonella foenum-graecum L*., Fenugreek, Leydig cells, Male reproduction

## Abstract

The objective of our *in vitro* study was to quantify the biochemical profile where the total polyphenol, flavonoid and phenolic acid content was determined. The antioxidant potential of microgreen extract from *Trigonella foenum-graecum L*., was measured molybdenum reducing power assay. Specifically, the study assessed parameters such as metabolic activity (AlamarBlue^TM^ assay), membrane integrity (CFDA-AM assay), mitochondrial potential (JC-1 assay), as well as reactive oxygen species generation (NBT assay). In addition, the steroid hormone release in TM3 murine Leydig cells after 12 h and 24 h exposures were quantified by enzyme-linked immunosorbent assay. The gained results indicate the highest value in total flavonoid content (182.59±2.13 mg QE) determination, supported by a significant (108.25±1.27 mg TE) antioxidant activity. The effects on metabolic activity, cell membrane integrity, and mitochondrial membrane potential were found to be both time- and dose-dependent. Notably, a significant suppression in reactive oxygen species generation was confirmed at 150, 200 and 250 μg/ml after 24 h exposure. In addition, progesterone and testosterone release was stimulated up to 250 μg/ml dose of Trigonella, followed by a decline in both steroid production at 300 and 1000 μg/ml. Our results indicate, that Trigonella at lower experimental doses (up to 250 μg/ml) may positively affect majority of monitored cell parameters in TM3 Leydig cells. Overleaf, increasing experimental doses may negatively affect the intracellular parameters already after 12 h of *in vitro* exposure.

## Introduction

Numerous reports suggest that several human diseases are increasingly prevalent compared to previous years. At the same time, they’re more progressive and their effects are more significant. This phenomenon is attributed to dynamic changes in lifestyles, eating habits, daily stress, polluted environment, and extensive usage of synthetic chemicals in food processing and agriculture [1,2]. Overleaf, the consumption of plant-based food, which is rich in various types of biological active molecules, phytonutrients, vitamins, minerals, fibres, or amino acids has increased rapidly in recent years. Many of these compounds possess antioxidant, immunomodulatory, and anti-carcinogenic properties that can significantly affect human and wildlife health [3,4]. Microgreens, which are miniature form of herbal plants, have gained significant popularity among the general population. They represent not only a modern culinary trend but are also increasingly utilized for health protection in various forms, including liquid extracts, solid preparations, nutritional supplements, and fresh consumption [5–7]. *Trigonella foenum-graecum L*., (family *Fabaceae*) is an annual, self-pollinated leguminous plant that is frequently used in the prevention and treatment of a broad spectrum of diseases, including cardiovascular diseases, hypercholesterolemia, hyperglycaemia, liver disorders and many others [8,1]. Its therapeutic effects can be attributed to a diverse profile of bioactive substances such as alkaloids (trigonelline, carpaine), phenols, flavonoids (quercetin, orientin), tannins, steroidal saponins (diosgenin, tigogenin), coumarin, and numerous secondary metabolites. Besides that, proteins, free amino acids (lysine, arginine), lipids, vitamins (A, B1, C), minerals (calcium, sodium), galactomannan fibres enhance its capacity to support overall health [9–11]. In addition, assessment of the nutritional quality and mutual ratios of biologically active molecules have confirmed significantly higher concentrations of bioactive compounds in *Trigonella* microgreens compared to their mature counterparts [12,13]. Due to the unique profile of Trigonella, the antioxidant [14], anti-inflammatory [15], anti-tumor [16] properties are well-known. However, there is some ambiguities regarding to the it’s effect on male reproductive system, and the mechanism of action is not fully understood. Kaur *et al*. [17] declare an improvement in mouse sperm parameters such as progressive motility and motility as well as increased sperm concentration. A positive effect of Trigonella aqueous extract was observed on sperm concentrations, sperm vitality and testosterone secretion [18]. In addition, Clemesha *et al*. [19] noted a significant increase in testosterone production and reported that Trigonella seeds contain a significant amount of diosgenin, which is a precursor of sex steroid hormones. On the other hand, Singh *et al*. [20,21] observed degenerative changes in the histoarchitecture of the testes, followed by a decrease in sperm concentrations, sperm viability and testosterone secretion. Current studies mostly describe only the impact on the production of male sex hormones, without detailed information regarding to the changed morphological and functional parameters of Sertoli cells or Leydig cells. Detailed information about molecular aspects and cellular processes *in vitro* could definitely clarify the impact of Trigonella on overall reproductive health in males. Therefore, the present *in vitro* study aims to characterize the biochemical profile and antioxidant potential of *Trigonella foenum-graecum L*. microgreens, as well as to evaluate their effect on cellular parameters, such as mitochondrial metabolism, membrane integrity, and mitochondrial membrane potential. In addition, the pro-oxidant and antioxidant efficacy that may influence steroid hormone release in murine Leydig cells was also examined.

## Materials and Methods

### Chemicals

We list chemicals and kits that were used solely for experimental analyses: AlamarBlue^TM^ reagent (AB; CAT. No. DAL1025), nitro-blue tetrazolium chloride (NBT; CAS No. 298-83-6), and dimethyl sulfoxide (DMSO; purity: ≥99.9 %, CAS No. 67-68-5), Dulbeccos’s phosphate-buffered saline (DBPS, D5773), Folin-Ciocalteu’s phenol reagent (CAT. No. F9252), Trolox (purity: ≥98 %, CAS No. 53188-07-1), aluminium chloride (CAS No. 7446-70-0), Dulbecco’s Modified Eagle Medium/Nutrient Mixture F-12 (CAS No. D9785), L-glutamine (CAS No. 56-85-9), fetal bovine serum (CAS. No. F7524) were purchased from Sigma Aldrich, Inc. (St. Louis, MO USA). JC-1 assay kit (CAT. No. T3168) and 5-carboxyfluorescein diacetate, acetoxymethyl ester (CFDA-AM; CAT. No. C1354) were obtained from Thermo Fisher Scientific, Inc (Watham, MA, USA). The testosterone ELISA kit (CAT. No. K00234), and the progesterone ELISA kit (CAT. No. K0025) were supplied by Dialab s.r.o. (Wiener Neudorf, Austria). Potassium hydroxide (KOH; purity: min. 85 %, CAS No. 1310-58-3), potassium acetate (C_2_H_3_O_2_K; purity: min. 99 %, CAS No. 127-08-2), sodium carbonate (Na_2_CO_3_; CAS No. 497-19-8), monopotassium phosphate (KH_2_PO_4_; purity: min. 99 %, CAS No. 16788-57-1), sulfuric acid (H_2_SO_4_; purity: min. 96 %, CAS No. 7664-93-9), ammonium heptamolybdate (NH_4_)_6_Mo_7_O_24_; purity: min. 98–100 %, CAS No. 12054-85-2), hydrochloric acid (HCl; CAS No. 7647-01-0), sodium hydroxide (NaOH; purity: min. 96 %, CAS No. 1310-73-2), ethanol (EtOH; purity: min. 96 %, CAS No. L00417) were purchased from CentralChem s.r.o. (Bratislava, Slovak Republic).

### Microgreens collection and processing

In this study, microgreens of *Trigonella foenum-graecum L*. were supplied by the local producer Microgreens s.r.o. (Bratislava, Slovak Republic). Trigonella seeds were germinated and cultivated in plastic trays in a phyto-chamber under a 16/8 h day/night regime at 22–24 °C, with relative air humidity fluctuating between 45–50 %. Specific details regarding the growth substrate and other cultivation conditions are not disclosed to maintain compliance with corporate confidentiality. The entire cultivation process was realized by Microgreens s.r.o., and the appropriate amount of sample was transferred to the AgroBioTech Research Centre (Slovak University of Agriculture in Nitra, Nitra, Slovak Republic). After fresh harvesting of Trigonella microgreens, the samples were dried at laboratory temperature, mechanically ground, and weighed in quantities corresponding to the number of analyses [22]. Additional processing of microgreens was performed depending on the type of analysis, as described below.

### Biochemical and antioxidant analysis of Trigonella extract

In brief, one gram of *Trigonella foenum-graecum L*. microgreens was weighed into 50 ml conical tubes and extracted with 10 ml of 80 % (v/v) ethanol (EtOH) during constant shaking at laboratory temperature for 12 h. Afterwards, the sample was centrifuged (9000 rpm, 4 °C, 5 min), and the supernatant was collected, and filtered by the PVDF syringe filter (0.45 μm). The extraction procedure was repeated twice, and finally, the two supernatants were polled to a final volume of 10 ml [22].

#### The total polyphenol content (TPC)

The total polyphenol content (TPC) was evaluated by the Folin-Ciocalteu’s phenol reagent (Sigma-Aldrich, St. Louis, MO, USA), as described previously by [23] with slight modifications. Briefly, 100 μl of Trigonella microgreens extract was mixed with the same volume of Folin-Ciocalteu’s phenol reagent, 1000 μl of 20 % (v/v) sodium carbonate (NA_2_CO_3_), and 8.8 ml of distilled water. Subsequently, the final mixture was incubated in the dark for 30 min. The absorbance was measured using a Jenway 6405 UV/VIS spectrophotometer (Fischer Scientific Inc.) at a 700 nm wavelength. TPC were expressed on a dry weight (d.w.) basis as mg of gallic acid equivalent (GAE) per gram d.w. of the sample [24].

#### The total flavonoid content (TFC)

The TFC was evaluated according to [25] with a slight modification of this method. In brief, the mixture of 0.5 ml of *Trigonella* microgreens extract, 0.1 ml of a 10 % (w/v) ethanolic solution of aluminum chloride (AlCl_3_), and the same amount of 1 M potassium acetate (C_2_H_3_O_2_K) was subsequently diluted in 4.3 ml of distilled water. Afterwards, the final mixture was incubated in the dark for 30 min. The absorbance was measured using a Jenway 6405 UV/VIS spectrophotometer (Fischer Scientific Inc.) at a 415 nm wavelength. TFC were expressed on a d.w. basis as mg of quercetin equivalent (QE) per gram d.w. of the sample [24].

#### The total phenolic acid content (TPAC)

For the evaluation of TPAC, a modified method of [25] study was used. Briefly, 0.5 ml of Trigonella microgreens extract was mixed with the same volume of 0.5 M hydrochloric acid (NaOH), and the Arnova reagent consisted of 10 % Na_2_MoO_4_, and 10 % NaNO_2_. In addition, 0.5 ml of 1 M sodium hydroxide (NaOH) (w/v) was supplemented, and subsequently diluted in the same amount of distilled water. The absorbance was measured using a Jenway 6405 UV/VIS spectrophotometer (Fischer Scientific Inc.) at a 490 nm wavelength. TPAC were expressed on a d.w. basis as mg of caffeic acid equivalent (CAE) per gram d.w. of the sample [24].

#### Antioxidant capacity (AC) of Trigonella extract

Molybdenum reducing power (MRP) was used for the determination of antioxidant activity of experimental extract, according to [27], with a slight modification. Firstly, the mixture of Trigonella’s extract, monopotassium phosphate (KH_2_PO_4_; 0.1 M), sulfuric acid (H_2_SO_4_; 1 M), ammonium heptamolybdate (NH_4_)_6_Mo_7_O_24_; 0.1 M), and distilled water was incubated for 2 h at 90 °C and, afterward, immediately cooled. The absorbance of the mixture was measured using a Jenway 6405 UV/VIS spectrophotometer (Fischer Scientific Inc.) at a 700 nm wavelength. The results were expressed as mg/g Trolox equivalents (TE) per d.w. of the sample.

### Cell culture

A cell line of murine TM3 Leydig cells (#CRL-1714TM) purchased from ATCC (American Type Culture Collection Ltd, Manassas, VA, USA) is a non-tumorigenic biological material gained from immature mouse testis. These cells are regularly used as appropriate models for the evaluation of toxic and non-toxic substances under *in vitro* conditions. Leydig cells used in this experiment were routinely cultured between 8^th^ and 25^th^ passages in 75 cm^2^ culture flasks (TPP AG, Trasadingen, Switzerland). They were passaged after reaching 75–80 % confluency and subcultured in a 1:10 to 1:50 ratio. TM3 cells were grown in Dulbecco’s Modified Eagle Medium/Nutrient Mixture F-12 (DMEM/F12; Sigma-Aldrich) supplemented with 2.5 mM L-glutamine (Sigma-Aldrich), 2.5 % (v/v) heat-inactivated fetal bovine serum (FBS; Biochrom AG, Berlin, Germany), 5 % (v/v) horse serum (HS; Gibco-Life Technologies Ltd, Auckland, New Zealand), and 1 % penicillin/streptomycin solution (Sigma-Aldrich), and maintained at 37 °C in 5 % CO_2_ and 95 % saturated atmospheric humidity. Cells were regularly screened for microbial contamination by the PlasmoTest^TM^ (InvivoGen Inc, San Diego, CA, USA). The TM3 cells were seeded at a density of 20000 cells/cm^2^ on 96-well plates, which were pre-coated overnight with 0.1 % w/v of gelatin (Sigma-Aldrich) dissolved in physiologically buffered saline (PBS; Sigma-Aldrich).

#### In vitro treatment

Briefly, one gram of Trigonella microgreens was weighed into 50 ml conical tubes and extracted with 10 ml of 80 % (v/v) EtOH during constant shaking at laboratory temperature for 12 h in the dark. Prepared crude extract was subjected to evaporation (Stuart RE300DB rotary evaporator, Bibby Scientific Limited Inc, UK) under reduced pressure (vacuum pump KNF N838.1.2KT.45.18, Freiburg, Germany) at 40 °C [28,29]. Subsequently, the extract of experimental microgreen *Trigonella foenum-graecum L*. was dissolved in DMSO, adjusted to 2000 μg/ml, and diluted in cell culture media DMEM/F12 to the final concentrations: 10; 50; 100; 150; 200; 250; 300; and 1000 μg/ml. The *in vitro* exposure of TM3 Leydig cells was taken during 12 h and 24 h. The effect of Trigonella given at experimental doses was set up based on our previous study with different microgreen [30]. The concentration of DMSO solvent did not exceed 0.6 % (v/v), and as a negative control was included on each experimental plate.

### Metabolic activity assay

In order to assess the impact of *Trigonella foenum-graecum L*. microgreen on the metabolic activity of Leydig cells, the AlamarBlue^TM^ assay was used. This test measures the enzymatic degradation of a commercially available resazurin solution, which is used to evaluate dehydrogenase activity in exposed cells [31,29]. In brief, after respective exposures (12 h and 24 h), wells were aspirated, cells were washed by DBPS, and DMEM/F12 containing 5 % (v/v) AlamarBlue reagent was applied. After 40 min incubation at 37 °C, 95 % atmospheric humidity, and 5 % CO_2_, the fluorescence at 530/590 nm wavelengths was measured using a microplate reader GlomaxMulti+ (Promega Corporation Ltd,, Madison WI, USA). The data obtained from the experimental groups were expressed as % of the control.

### Cell membrane integrity assay

5-carboxyfluorescein diacetate, acetoxymethyl ester (CFDA-AM) is metabolized to the polar, fluorescent carboxyfluorescein by the nonspecific enzymatic activity of esterase in living cells. This conversion reflects potential alterations in membrane integrity [32,33]. In brief, after respective treatments (12 h and 24 h), cell culture media was removed from the wells, and TM3 cells were washed with DPBS, and cultured with fresh DMEM/F12 containing CFDA-AM at a final concentration of 4 μM. After 40 min incubation at 37 °C, 95 % atmospheric humidity, and 5 % CO_2_, the fluorescence at 485/530 nm wavelengths was measured using a microplate reader GlomaxMulti+ (Promega Corporation Ltd.). The data obtained from the experimental groups were expressed in % of the control.

### Mitochondrial membrane potential (ΔΨm) assay

The mitochondrial membrane potential was determined through the commercially available JC-1 assay kit. The principle covers the process of fluorescent cationic dye binding to the mitochondria and adjusting its fluorescent features depending on its aggregation. Concretely, functional mitochondria with high *ΔΨm* emit red fluorescence, while disrupted mitochondria with low *ΔΨm* exhibit green fluorescence [34]. After respective treatments (24 h) of Leydig TM3 cells by *Trigonella*, 1×10^6^ cells were transferred into Eppendorf tubes. The cell suspension was diluted to 100 μl with DPBS and immediately stained with the JC-1 working solution. TM3 cells were incubated at 37 °C, 95 % atmospheric humidity, and 5 % CO_2_ for 20 min. Then, the solution was removed, and cells were washed with DPBS. After the transportation into a 96-well plate, the fluorescence intensity was measured using a microplate reader GlomaxMulti+ (Promega Corporation Ltd.). The resulting *ΔΨm* is expressed as the ratio of JC-1 complexes to JC-1 monomers (red/green ratio) [35].

### Intracellular levels of superoxide radicals (•O2)

The principle of the nitroblue-tetrazolium assay (NBT) covers the reaction between membrane permeable yellow-colored 2,2′-bis(4-nitro-phenyl)-5,5′-diphenyl-3,3′-dimethoxy-4,4′-diphenylene) diterazolium chloride and •O_2_ leading to the formation of blue formazan deposits [36]. In brief, after exposure of TM3 cells to *Trigonella* (12 h and 24 h), the NBT working solution containing nitroblue-tetrazolium salt dissolved in DMEM/F12 with 1.5 % DMSO was applied. Subsequently, cells were incubated in 37 °C, 95 % atmospheric humidity, and 5 % CO_2_ for 3 h. The reaction was stopped by 2 M KOH dissolved in DMSO. The optical density was quantified with the microplate reader Multiscan FC (Thermo Fisher Scientific Inc, Waltham, MA, USA) at set wavelengths of 620 nm and 570 nm. The data obtained from the experimental groups were expressed as % of the control.

### Steroid hormone assay

The Enzyme-Linked Immunosorbent assay (ELISA) is a technique to detect the presence of antigens in biological samples. Specifically, this method relies on antibodies to detect a target antigen using highly specific antibody-antigen interactions (ELISA manual). After the respective exposure (12 h and 24 h) of Leydig cells to *Trigonella*, cell culture media was aspirated from each well and transferred to the marked Eppendorf tubes. Due to the remnant’s removal of the cellular elements, centrifugation was carried out at 3000 rpm for 10 min at 4 °C. Subsequently, supernatants were stored at −80 °C until steroid hormone quantifications. Commercially available ELISA kits intended for progesterone and testosterone determination were purchased from Dialab. The procedure was carried out according to the manufacturer’s instructions. Briefly, an aliquot of samples and calibrators containing the antigens to be quantified is added to and allowed to bind with a solid-phase antibody (microplate wells). Afterwards, an enzyme-labeled antibody was added, and a solid-phase complex was formed. Unbound antibodies were washed away by a washing solution, and enzyme substrate was added. After 15 min of incubation in the dark, the reaction was finished by stop solution. The absorbance was measured using an ELISA microplate reader at 450 nm wavelength (Multiscan FC, ThermoFisher Scientific Inc.). The amount of the final product is proportional to the amount of progesterone or testosterone in the samples. The data obtained from the measurements were expressed as % of the control. The intra- and inter-assay variability and sensitiveness for the selected steroid hormones are summarized in Table 3.

### Data and statistics

Data representing independently repeated experiments (at least three independent repetitions, unless stated otherwise) were combined and used for further analysis. Collected data passed through Shapiro-Wilk’s normality test, followed by analyses of descriptive characteristics (min., max., mean, standard error of the mean, etc.). One-way analysis of variance (ANOVA), followed by Dunnett’s multiple comparison tests, was used to examine differences between the experimental and control groups. The results were expressed as the mean ± standard deviation (SD). P-values equal to or lower than 0.05 were considered statistically significant. All statistical analyses were performed using GraphPad Prism 6.01 (GraphPad Software Inc., San Diego, CA, USA).

## Results

### Biochemical profile and antioxidant capacity of Trigonella foenum-graecum L. microgreens

The total polyphenol, flavonoid, and phenolic acid contents in *Trigonella* microgreen extract was determined as follows: The Folin-Ciocalteu method was used for TPC in the experimental sample, where the level was estimated at 60.87 (±14.83) mg GAE/gram d.w. For the TFC determination, the measured level was 182.59 (±2.13) mg QE/gram d.w. Further analysis of TPAC confirmed a value of 40.96 (±1.63) mg CAE/gram d.w. In addition, the MRP method revealed that free-radical scavenging potential reached the level of 108.25 (±1.27) mg TE/gram d.w. The obtained data are summarized in Table 1.

### Changes in metabolic activity of Leydig TM3 cells induced by Trigonella extract in vitro

**Fig. 1:**
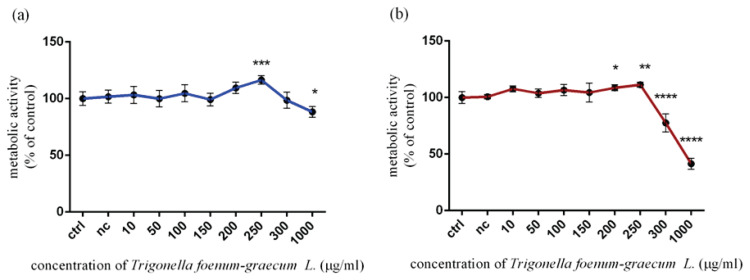
Metabolic activity of TM3 Leydig cells treated with various concentrations of *Trigonella foenum-graecum L*. during (**a**) 12 h and (**b**) 24 h period *in vitro*. ctrl – control group (not treated), nc – negative control group (DMSO). Each bar represents the mean (± SD) optical density percent of the control group and microgreen’s extract treated groups. The level of statistical significance was established at ****(p<0.0001); ***(p<0.001); **(p<0.01), and *(p<0.05). Statistical differences are indicated by an asterisk.

The *in vitro* impact of *Trigonella foenum-graecum L*. at different experimental doses was evaluated by an AlamarBlue assay with regard to metabolic activity of exposed cells. Based on the results, the cytotoxic impact was confirmed to be dose- and time-dependent. As shown in Figure 1a the metabolic activity was significantly increased (p<0.001) at 250 μg/ml (116.40±3.80 %). On the other hand, the highest experimental concentration (1000 μg/ml) of *Trigonella foenum-graecum L*. caused a significant reduction (p<0.05) in metabolic activity (88.27±4.67 %) after 12 h. In the case of 24 h exposure (Fig. 1b), a significant (p<0.05; p<0.01) stimulation of metabolic activity was observed at 200 μg/ml (108.60±2.64 %) and 250 μg/ml (111.30±2.32 %). Overleaf, the significant cytotoxic effect was identified at 300 μg/ml (p<0.0001; 77.56±8.03 %) and 1000 μg/ml (p<0.0001; 41.46±4.83 %) compared to the control group (100.00±4.93 % vs. 8.37 %).

### Changes in membrane integrity of Leydig TM3 cells induced by Trigonella extract in vitro

**Fig. 2:**
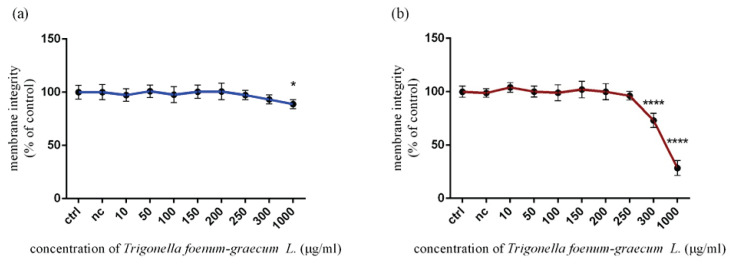
Membrane integrity of TM3 Leydig cells treated with various concentrations of *Trigonella foenum-graecum L*. during (**a**) 12 h and (**b**) 24 h period *in vitro*. ctrl – control group, (not treated), nc – negative control group (DMSO). Each bar represents the mean (± SD) optical density percent of the control group and microgreen’s extract treated groups. The level of statistical significance was established at ****(p<0.0001), and *(p<0.05). Statistical differences are indicated by an asterisk.

Cell membrane integrity was another parameter which was measured using a carboxyfluorescein diacetate (CFDA-AM) assay. According to the obtained results, cell membrane integrity was not significantly affected during 12 h exposure, except the highest experimental dose (88.80±4.22 %) of microgreen extract followed by significant changes (p<0.05) showed in (Fig. 2a). On the other side, Figure 2b presented significant (p<0.0001) defects in cell membrane compactivity, which were recorded at 300 μg/ml (73.08±6.55 %) and 1000 μg/ml (28.45±7.03 %) after 24 h exposure *in vitro*. All experimental doses were compared to the untreated (control) cells (100.00±5.16 %).

### Changes in ΔΨm of Leydig TM3 cells induced by Trigonella extract in vitro

For the detailed look on the mitochondrial behaviour in Leydig cells, the JC-1 assay was used. Due to the weak changes recorded during 12 h exposure in the previous parameters, the *ΔΨm* was quantified only after 24 h of exposure. Gained results revealed significant (p<0.05) changes at 150 μg/ml and 200 μg/ml, followed by significant (p<0.01) stimulation at 250 μg/ml of Trigonella extract. Inversely, the JC-1 assay confirmed significant (p<0.01) disrupting effects and decreased mitochondrial membrane potential at 300 μg/ml and 1000 μg/ml (p<0.0001) compared to control group. The results are summarized in Table 2.

### Effects of Trigonella foenum-graecum L. doses on superoxide radical production in TM3 Leydig cells in vitro

**Fig. 3:**
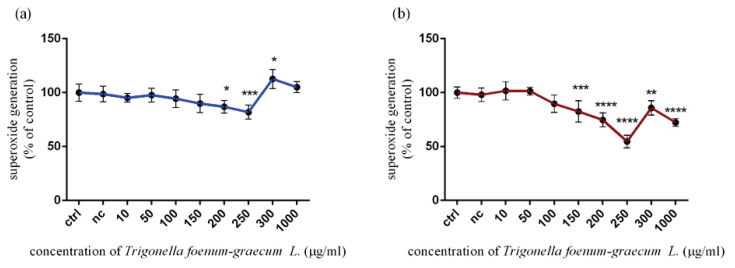
Superoxide radical production in TM3 Leydig cells treated with various concentrations of *Trigonella foenum-graecum L*. during (**a**) 12 h and (**b**) 24 h period *in vitro*. ctrl – control group (not treated), nc – negative control group (DMSO). Each bar represents the mean (± SD) optical density percent of the control group and microgreen’s extract treated groups. The level of statistical significance was established at ****(p<0.0001); ***(p<0.001); **(p<0.01), and *(p<0.05). Statistical differences are indicated by an asterisk.

The results gained from the NBT assay described in Figure 3a confirmed a significant (p<0.05; p<0.001) decrease in superoxide radical production at 200 μg/ml and 250 μg/ml (86.90±5.97 %; 81.98±6.57 %) of *Trigonella foenum-graecum L*. after 12 h *in vitro* cultivation. On the contrary, 300 μg/ml of microgreen extract significantly (p<0.05) enhanced the superoxide radical level (112.60±8.81 %), followed by the same trend at 1000 μg/ml without significant changes compared to the control group (100.00±7.97 %). Results presented in Figure 3b showed a similar tendency in superoxide radical production after 24 h exposure. The antioxidant potential of Trigonella microgreens with significant (p<0.001; p<0.0001) impact was confirmed at 150 μg/ml (82.62±9.77 %), 200 μg/ml (74.73±6.40 %), and 250 μg/ml (54.64±5.84 %). Besides that, significant changes (p<0.001; p<0.01) were also recorded at 300 μg/ml and 1000 μg/ml of Trigonella exposure compared to the control group (100.00±2.10 %).

### Effects of Trigonella foenum-graecum L. doses on steroid hormone release in TM3 Leydig cells in vitro

#### Progesterone release

**Fig. 4:**
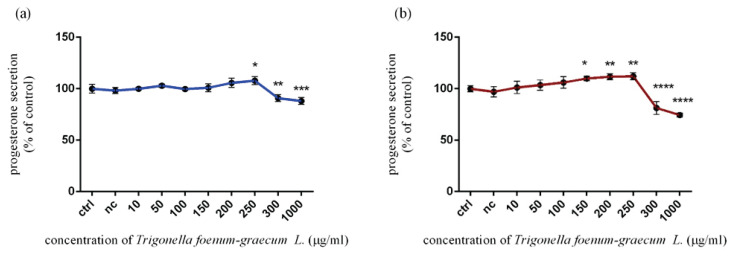
Progesterone release in TM3 Leydig cells treated with various concentrations of *Trigonella foenum-graecum L*. during (**a**) 12 h and (**b**) 24 h period *in vitro*. ctrl – control group, (not treated), nc – negative control group (DMSO). Each bar represents the mean (± SD) optical density percent of the control group and microgreen’s extract treated groups. The level of statistical significance was established at ****(p<0.0001); ***(p<0.001); **(p<0.01), and *(p<0.05). Statistical differences are indicated by an asterisk.

In the case of 12 h exposure, Figure 4a indicates a non-significant change through the panel of applied concentrations of microgreen extract up to 250 μg/ml. This concentration started to stimulate progesterone release (107.90±3.77 %) at a significant level (p<0.05). Overleaf, the highest doses (300 μg/ml and 1000 μg/ml) of *Trigonella foenum-graecum L*. caused a significant (p<0.01; p<0.001) reduction in progesterone release *in vitro*. The progesterone level was set at 90.85±3.29 % and 88.10±3.31 %, respectively. After 24 h exposure (Fig. 4b), a significant (p<0.05) enhancement of progesterone release was recorded at 150 μg/ml (109.90±2.30 %), followed by significant (p<0.01) stimulation by 200 μg/ml (111.70±2.80) and 250 μg/ml (112.00±3.39 %) of microgreen extract. Conversely, the actions of 300 μg/ml (81.28±6.23) and 1000 μg/ml (74.40±1.82 %) caused a progressive decline in this parameter with a significant (p<0.0001) impact. All the data were compared to the control group (100.00±3.08 %).

#### Testosterone release

**Fig. 5:**
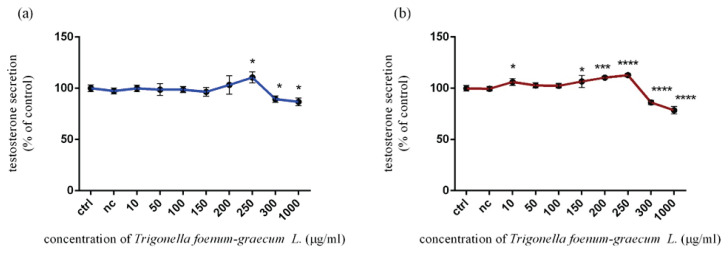
Testosterone release in TM3 Leydig cells treated with various concentrations of *Trigonella foenum-graecum L*. during (**a**) 12 h and (**b**) 24 h period *in vitro*. ctrl – control group (not treated), nc – negative control group (DMSO). Each bar represents the mean (± SD) optical density percent of the control group and microgreen’s extract treated groups. The level of statistical significance was established at ****(p<0.0001); ***(p<0.001), and *(p<0.05). Statistical differences are indicated by an asterisk.

As seen in Figure 5a, the experimental dose (250 μg/ml) of *Trigonella foenum-graecum L*. significantly (p<0.05) enhanced testosterone release (110.5±5.39 %), followed by a significant decrease at 300 μg/ml (89.48±3.14 %) and 1000 μg/ml (86.81±3.76 %) after 12 h of *in vitro* exposure. As shown in Figure 5b, the prolonged time of cultivation (24 h) caused a moderate growth in testosterone release at 10 μg/ml (106.20±3.35 %) and 150 μg/ml (106.70±5.86 %) of microgreen extract treatment. The same trend with a significant (p<0.001; p<0.0001) impact continued gradually at 200 μg/ml (110.40±1.38 %) and 250 μg/ml (112.80±1.83 %). In the case of the highest doses (300 μg/ml and 1000 μg/ml), a significant (p<0.0001) inhibition was recorded. The level of testosterone secretion was established at 86.16±2.16 % and 78.53±3.60 % in comparison to the control group (100.00±2.64 %).

## Discussion

The identification of new sources of biologically active compounds in the human diet that have proven potential to protect individual health and promote male reproductive health is a significant focus of investigation in recent decades. Our *in vitro* study characterized the biochemical profile of *Trigonella foenum-graecum L*. microgreens and assessed its overall antioxidant capacity. In addition, the potential impact of experimental extract on different functional parameters such as metabolic activity, cell membrane integrity, mitochondrial membrane integrity, superoxide production and steroid hormone secretions were investigated. Our study established the total polyphenol content (TPC) at 60.87 (±14.83) mg GAE/g d.w., while the total flavonoid content (TFC) was estimated at 182.59 (±2.13) mg QE/g d.w. Further analyses of phenolic acid content (TPAC) identified the level at 40.96 (±1.63) mg CAE/g d.w. In addition, the results from MRP assay showed a significant antioxidant potential measured at 108.25 (±1.27) mg TE/g d.w. Grygorieva [37] also quantified the overall content of bioactive components such as TPC or TFC in methanolic extract of *Trigonella foenum-graecum L*. in a recent study. The authors compared individual parameters in seeds, stems, leaves, and microgreens of Trigonella. The results clearly showed that TPC (63.91±4.55 %), TFC (19.07±0.20 %) and TAA (total ascorbic acid (0.33±0.03 %) were significantly higher in microgreens compared to seeds, leaves, and stems of their mature counterparts. The presented study also examined the antioxidant activity of *Trigonella*. Out of all the examined samples, microgreens extract exhibited the highest percentage of antioxidant activity compared to all methanolic extracts from seed, leaf, and stem of adult plants. In summary, *Trigonella* microgreens are significantly more abundant in polyphenols, flavonoids, or phenolic acids than their mature counterparts, which is directly related to the eminent overall antioxidant capacity of *Trigonella* microgreens. Based on our quantification of the TPC, TFC and TPAC in the experimental Trigonella’s extract, and in accordance with the findings of Singh [21], we may conclude that high levels of these bioactive molecules, namely diosgenin, gitogenin and especially trigonelline, have a positive effect on ovarian functions as well as on reproductive functions in males. Steels [38] reported, that diosgenin is supposed to be a precursor of many sex hormones. Recent studies carried out by Sirotkin [39] and Sorrenti [40] also report that Trigonella and diosgenin can be applied in reproductive dysfunctions and point to a stimulatory action on ovarian follicullogenesis. Moreover, evaluation of diosgenin’s effect on porcine granulosa cells demonstrated its ability to promote ovarian cell turnover. On the other hand, in cultured human ovarian cancer cells, a Trigonella extract containing diosgenin increased apoptosis and reduced viability of exposed cells [41].

Although several studies have recently addressed the impact of Trigonella on male reproductive potential [42,43], the effects of *Trigonella* microgreens on male reproductive cells *in vitro* have not yet been investigated. When considering the currently accumulated data regarding Trigonella’s impact on reproductive health in males, we can note positive effects on the majority of reproductive parameters [44,45]. Conversely, excessive concentrations [46] have shown negative impact on the weight of the testis, damage of seminiferous tubules, and disruptions in the interstitial space of the testis. In addition, testosterone concentrations were significantly reduced (more than 50 % lower) compared to the control group. Focusing specifically on the *in vitro* effects of *Trigonella* microgreens doses, we can reference a study performed by [45]. Authors examined the impact of *Trigonella* on murine Leydig cell viability using the MTT assay. Their results revealed a potential to stimulate cell viability at 50 μg/ml of *Trigonella* extract after 24 h exposure. The impact on cell viability was also examined by [47], who evaluated *Trigonella*’s effect on rats’ spermatozoa after 30 days of oral administration. The gained results confirmed that 15 mg/kg body weight positively affect spermatozoa viability. Our *in vitro* study is conceptually linked to studies by the above-mentioned authors. *Trigonella* microgreens administered at doses up to 250 μg/ml increased the viability of TM3 Leydig cells, while higher concentrations (300 μg/ml and 1000 μg/ml) caused significant inhibition of this parameter. Our *in vitro* study also evaluated other parameters, such as cell membrane integrity, and mitochondrial membrane potential. The results showed a significant decrease in cell membrane integrity of exposed TM3 cells at 300 μg/ml and 1000 μg/ml of *Trigonella* microgreens extract, while a decrease in mitochondrial membrane potential was detected at concentrations ranging from 150 μg/ml to 1000 μg/ml. A notable inhibition of mitochondrial membrane potential caused by *Trigonella* extract was reported by [48]. Their results showed that the application of experimental dose of 50 μg/ml of *Trigonella* for 12 h significantly affected mitochondrial membrane potential in breast cancer cells (MCF-7). Li [49] quantified mitochondrial membrane potential using the JC-1 method, which was also used in our studies. Individual biologically active substances from *Trigonella* were applied on 3T3-L1 adipocytes for 48 h. The results confirmed a significant decrease in mitochondrial membrane potential and indicated damage of mitochondrial functions. Besides that, authors assessed the effect of polyphenolic compounds isolated from *Trigonella* on reactive oxygen species (ROS) production after 48 h of exposure. The results of the DCFH-DA analysis clearly showed that individual biologically active compounds from *Trigonella* possess significant potential to inhibit ROS production. This tendency was also observed in our study, when higher doses applied inhibited ROS generation after 24 h of exposure. The suppression of ROS production was also confirmed by a previous study [50]. In that study, overproduction of ROS was induced using 0.5 mM ethanol for 24 h. Subsequent application of *Trigonella foenum-graecum L*. suppressed this production, with the highest experimental dose of *Trigonella* (25 μg/ml) demonstrating significant inhibition. Based on the confronted results, we can conclude that *Trigonella foenum-graecum L*. may significantly inhibit ROS generation, and thus suppress the onset of oxidative stress in exposed cells *in vitro*. Our results suggest that the rich and high content of biologically active substances in Trigonella microgreens has a strong antioxidant potential. The results gained during realisation of our *in vitro* experiment revealed fundamental changes in cellular parameters of TM3 Leydig cells which may directly affect the process of steroidogenesis, initiate a change in the activity of steroidogenic enzymes, or the onset of cell apoptosis. Several previous studies have identified *Trigonella foenum-graecum L*. as an herb with testosterone-boosting potential [51,8,1]. Therefore, the aim of our study was to investigate intracellular changes in the used experimental model TM3 Leydig cells after 24 h and 48 h, which could be related to changes in progesterone and testosterone secretion. The results obtained confirmed the significant potential of *Trigonella* microgreens to stimulate progesterone and testosterone release *in vitro* after 24 h and 48 h of incubation. Wilborn [52] and Wankhede [53] declare an increase in steroid hormone production, which has been stimulated by specific phytochemicals found in *Trigonella*. At the same time, they demonstrated the potential to inhibit aromatase and 5-alpha-reductase. *In vitro* studies have confirmed that biologically active compounds such as glycosides, saponins, or sapogenins exhibit significant androgenic and anabolic potential. In case of the regulation of ovarian functions, cell membrane integrity, mitochondrial activity, cell proliferation and sex hormone secretion play an important role. Grzesiak [54] pointed out an increase in ovarian cell proliferation and confirmed the stimulation of the biosynthesis of sex hormones, specifically estradiol. Swaroop’s [55] study confirmed, that lower Trigonella doses have significant effect on luteinizing (LH) and follicular stimulation (FSH) hormones, which stimulate the ovarian functions in females. Besides that, Abedinzade [56] pointed out changes in cholesterol levels, what is directly linked with the secretion of female sex-hormone such as 17β-estradiol. All of the above findings indicate that the regulation of ovarian functions, as well as changes in the cellular parameters of the male reproductive system are dependent on deviation in gonadotropins, secretions (LH, FSH), intraovarian factors and the content of biologically active molecules in Trigonella. These molecules can interfere with the endogenous action of hormones, and subsequently affect other levels of endocrine regulation. We are assumed, that bioactive molecules initiate changes in cholesterol levels, what may directly affect the secretion of steroid hormones themselves, including through deviation in the activity of steroidogenic enzymes such as 3β-HSD or StAR protein [55,21].

Based on the currently available scientific papers, we may report, that information about the effect of *Trigonella* microgreens doses on intracellular parameters and the physiological states of different cellular models is limited. Although our study provided new information about *in vitro* effect of Trigonella on mice Leydig cell parameters, the limiting factor is the absence of gene expression regarding to steroid enzymes [54,57]. In the future, it is necessary to carry out this screening, what could help to clarify the specific the action of bioactive molecules in Trigonella, and subsequently determined the impact on reproductive functions in males and females. If the mechanism of action will be known in detailed, it is possible to use the achieved results in reproductive biology and medicine. The ability of Trigonella to affect the secretion of gonadotropins, to stimulate steroidogenesis, to inhibit ROS generation, and encourage antioxidant defence indicates the potential to use it in the prevention of reproductive aging, stimulation of libido, or inhibition of the onset of andropause or testosterone deficiency syndrome [58,59]. In addition, it is possible to use a rich nutritional profile in ruminant feed and, thanks to the high content of biologically active substances, support the overall health of the individual [60].

## Conclusions

The results of our *in vitro* analyses indicated significant changes in intracellular processes and essential parameters that can contribute to the overall support of the male reproductive system after the application of *Trigonella foenum-graecum L*. microgreens.

Gained results confirmed, Trigonella microgreen given at the lower concentrations could clearly stimulate the metabolic activity, cell membrane integrity and steroid hormone secretion after 24 h and 48 h exposure. In addition, experimental doses up to 250 μg/ml may significantly suppress ROS generation. Overleaf, increasing doses, mainly 300 and 1000 μg/ml may cause dramatic damages in monitored parameters, followed by significant inhibition in progesterone and testosterone secretion. This is one of the few studies that, in addition to conducting phytochemical screening of Trigonella microgreens, carefully examines the molecular changes in exposed Leydig cells *in vitro*.

## Figures and Tables

**Table 1:** Biochemical parameters and antioxidant activity of *Trigonella foenum-graecum L*. microgreens.

Parameter	Values (gram/d.w.)
*The total polyphenol content*	60.87 (±14.83) mg GAE
*The total flavonoids content*	182.59 (±2.13) mg QE
*The total phenolic acid content*	40.96 (±1.63) mg CAE
*Molybdenum reducing power*	108.25 (±1.27) mg TE

Abbreviations: Data are presented as means (± SD) from four independent measurements. GAE – gallic acid equivalents; QE – quercetin equivalents; CAE – caffeic acid equivalents; TE – Trolox equivalents.

**Table 2:** Mitochondrial membrane potential (*ΔΨm*) of TM3 Leydig cells treated with various concentrations of *Trigonella foenum-graecum L*. during 24 h exposure *in vitro*.

*Trigonella* (μg/ml)	ctrl	sc	10	50	100	150	200	250	300	1000
**ΔΨm red/green ration**	0.20±0.01	0.21±0.02	0.21±0.01	0.21±0.01	0.22±0.02	0.23^*^±0.01	0.23^*^±0.02	0.24^**^±0.01	0.16^**^±0.01	0.13^****^±0.01

Abbreviations: ctrl – control group (not treated), nc – negative control group (DMSO).

Data are presented as mean ± SD. The level of statistical significance was established at ^****^(p<0.0001); ^**^(p<0.01), and ^*^(p<0.05). Statistical differences are indicated by an asterisk.

**Table 3:** Intra-assay, inter-assay variability and sensitiveness for the selected steroid hormones.

Hormone	Intra-assay variability (%)	Inter-assay variability (%)	Sensitivity
*Progesterone*	≤4.0	≤9.3	0.05 ng/ml
*Testosterone*	≤7.0	≤8.3	0.10 ng/ml
